# Monocytic Myeloid-Derived Suppressor Cells Inhibit Myofibroblastic Differentiation in Mesenchymal Stem Cells Through IL-15 Secretion

**DOI:** 10.3389/fcell.2022.817402

**Published:** 2022-02-17

**Authors:** Yin Celeste Cheuk, Shihao Xu, Dong Zhu, Yongsheng Luo, Tian Chen, Juntao Chen, Jiawei Li, Yi Shi, Yi Zhang, Ruiming Rong

**Affiliations:** ^1^ Department of Urology, Zhongshan Hospital, Fudan University, Shanghai, China; ^2^ Shanghai Key Laboratory of Organ Transplantation, Shanghai, China; ^3^ Institute of Clinical Science, Zhongshan Hospital, Fudan University, Shanghai, China

**Keywords:** MDSC (myeloid-derived suppressor cells), mesenchymal stem cells, myofibroblastic differentiation, IL-15, fibrosis

## Abstract

**Background:** Accumulating evidence indicates that mesenchymal stem cells (MSCs) are precursors of myofibroblasts, which play a vital role in renal fibrosis. The close interaction between MSCs and other immune cells regulates the development of multiple fibrosis-related diseases. However, the effect of myeloid-derived suppressor cells (MDSCs) on MSCs remains unexplored. Here, we investigated the effect of MDSCs on the myofibroblastic differentiation of MSCs.

**Methods:** MSCs were induced to undergo myofibroblastic differentiation with transforming growth factor beta 1 (TGF-β1). M-MDSCs and G-MDSCs were sorted by flow cytometry. Supernatants derived from MDSCs were administered to cultured bone marrow MSCs (BM-MSCs) undergoing TGF-β1-induced myofibroblastic differentiation. Myofibroblastic differentiation was evaluated by immunostaining. The expression of fibrosis-related genes was determined by quantitative PCR and western blot analysis. *In vitro*, M-MDSC supernatant or M-MDSC supernatant with interleukin (IL)-15 mAbs was administered following unilateral renal ischemia-reperfusion injury (IRI) to observe the myofibroblast differentiation of renal resident MSCs (RRMSCs) in a murine model.

**Results:** Myofibroblastic differentiation of MSCs was hindered when the cells were treated with MDSC-derived supernatants, especially that from M-MDSCs. The inhibitory effect of M-MDSC supernatant on the myofibroblastic differentiation of MSCs was partially mediated by IL-15-Ras-Erk1/2-Smad2/3 signaling. Treatment with M-MDSC supernatant ameliorated renal fibrosis and myofibroblastic differentiation in RRMSCs through IL-15. Additionally, M-MDSC supernatant increased M-MDSC infiltration in the kidney in a mouse IRI model. M-MDSC supernatant downregulated the adhesion and migration marker CD44 on the cell membrane of MSCs via IL-15.

**Conclusion:** M-MDSC-derived supernatant inhibited the TGF-β1-induced myofibroblastic differentiation of MSCs through IL-15. Our findings shed new light on the effect of MDSCs on myofibroblastic differentiation and adhesion of MSCs, which might provide a new perspective in the development of treatment strategies for renal fibrosis.

## Introduction

Mesenchymal stem cells (MSCs) are a type of multipotent stem cell of mesodermal origin that is derived from practically all tissue adventitial progenitor cells and pericytes (Ma et al., 2020). Because of the ability of these cells to differentiate into hematopoietic, liver, cardiac, endothelial, nerve, islet and several other cell types, MSCs provide a new strategy for the clinical treatment of numerous diseases (Regmi et al., 2019). However. MSCs are not always beneficial and protective (Basalova et al., 2020). Under the influence of profibrotic cytokines and signals, such as TGF-β1, Wnt10 (Cao et al., 2020), RhoA/Rock (Ke et al., 2019), and Smad3/Erk1/2 (Tang et al., 2018), MSCs can directly transdifferentiate into myofibroblasts. A large body of evidence suggests that myofibroblasts are crucial cells in the process of renal fibrosis, are highly heterogeneous and derive from resident fibroblasts, pericytes and bone marrow (BM)-derived cells (Volarevic et al., 2017). LeBleu et al. (2013) found that circulating BM-MSCs contributes to renal myofibroblasts. Kramann et al. (2015) reported that renal myofibroblasts were derived from kidney-resident MSC-like cells. In addition, kidney pericytes harbor an MSC population. By using single-cell RNA sequencing, Kuppe et al. (2021) profiled the transcriptomes of cells from healthy and fibrotic human kidneys to identify distinct subpopulations of pericytes as the main cellular sources of scar-forming myofibroblasts during human kidney fibrosis. Thus, circulating BM-MSCs, resident perivascular MSC-like kidney cells or pericytes have recently been regarded as major sources of myofibroblasts that drive kidney fibrosis. Transforming growth factor beta 1 (TGF-β1) is a key driving force in myofibroblastic differentiation. Therefore, it is critical to study how to inhibit TGF-β1-induced differentiation of MSCs into myofibroblasts to attenuate renal fibrosis.

In recent years, the interaction between MSCs and immune cells has been elucidated. The effect of MSCs on immune cells includes promoting monocyte polarization to anti-inflammatory M2 macrophages, suppressing the maturation of dendritic cells, and reducing the cytotoxicity of NK cells (Ma and Chan, 2016). Moreover, immune cells are capable of regulating the differentiation and function of MSCs. Ma et al. (2020) showed that macrophage-derived supernatants inhibited the adipogenic differentiation of human adipose-derived stem cells (hADSCs) *in vitro*, in which M1-sup was more potent due to higher expression of proinflammatory cytokines. Saldana et al. (2019) showed that factors secreted by proinflammatory and anti-inflammatory macrophages activated the immunomodulatory potential of MSCs, which was partially attributed to the priming effect of TNF-α and IL-10. Myeloid-derived suppressor cells (MDSCs) are innate cells that play a major role in immunosuppression and can be divided into two major groups in mice: granulocytic MDSCs (G-MDSCs; CD11b^+^Ly6G^+^Ly6C^low^) and monocytic MDSCs (M-MDSCs; CD11b^+^Ly6G^−^Ly6C^high^) (Li et al., 2021). G-MDSCs and M-MDSCs also have unique functional and biochemical characteristics. Studies have shown that G-MDSCs inhibit fibrosis in the kidney (Yan et al., 2020) and liver (Hochst et al., 2015), while G-MDSCs promote fibrosis in pulmonary hypertension (Lebrun et al., 2017). Other research has suggested that M-MDSCs inhibit fibrosis in liver disease (Wang et al., 2019) and promote pulmonary fibrosis by producing TGF-β1 (Fu et al., 2021). However, the mechanisms remain to be elucidated. Although there have been studies indicating that MSCs inhibit the differentiation of MDSCs into G-MDSCs by secreting prostaglandin E2 (PGE2) or inhibit the differentiation of M-MDSCs by secreting interferon (IFN)-β (Qi et al., 2020), whether MSC differentiation is affected by MDSCs or MDSC subtypes in the fibrotic process is still unknown.

Interleukin (IL)-15 is a pleiotropic cytokine that is involved in various biological activities. IL-15 mRNA expression has been detected in a wide range of tissues, both in hematopoietic and nonhematopoietic cells such as nerve cells, keratinocytes, fibroblasts and stromal cells (Yang and Lundqvist, 2020). In contrast to widespread IL-15 mRNA expression, mature IL-15 protein production is mainly limited to monocytes/macrophages and DCs (Yang and Lundqvist, 2020). IL-15 is known to be present both as secreted and membrane-bound forms. IL-15 induces the activation of Jak/STAT, PI3K, Src family tyrosine kinases, MAPKs and NF-kB in immune cells through IL-15Rα, as well as two coreceptors, IL-2Rβ (also known as IL-15Rβ) and the IL-2Rγ common chain (Beech et al., 2012). Previous studies have reported that IL-15 is associated with inducing the differentiation and proliferation of lymphocytes and natural killer (NK) cells (Patidar et al., 2016). IL-15 is also suggested to be negatively associated with fibrosis in the kidney (Devocelle et al., 2019), lung (Venkateshaiah et al., 2019), liver (Jiao et al., 2016) and pancreas (Manohar et al., 2018). Additionally, IL-15 could regulate adhesion-associated molecules on NK and T cells (Estess et al., 1999; Lin et al., 2016; Hydes et al., 2018). The effect of IL-15 on MSCs needs further study.

In this study, we examined the effect of MDSCs on the myofibroblastic differentiation of MSCs and the mechanisms involved. We found that M-MDSC-derived supernatant inhibited the TGF-β1-induced myofibroblastic differentiation of MSCs though IL-15 secretion. Exogenous M-MDSC supernatant treatment attenuated fibrosis and increased M-MDSC infiltration in a renal IRI model. Moreover, M-MDSC supernatant downregulated the adhesion and migration marker CD44 on the cell membrane of MSCs via IL-15. These findings expand our understanding of cellular interactions between MDSCs and MSCs, providing a new direction for the treatment of renal fibrosis.

## Materials and Methods

### Culture of Mesenchymal Stem Cells

The bone marrow MSCs of C57BL/6 mice were obtained from Cyagen Biosciences (Suzhou, China) and verified by examining the expression of the surface antigens CD29, CD44, CD117, Sca-1 and CD31 by using a FACS Aria III flow cytometer (Supplementary Figure S1A). The antibodies were purchased from Cyagen Biosciences (Suzhou, China). The cells were cultured in DMEM (Gibco, CA, USA) containing fetal bovine serum (FBS), l-glutamine, and a penicillin–streptomycin solution (Gibco, CA, USA) and were treated with or without TGF-β1 (PeproTech, USA), MDSC supernatant, IL-15 (PeproTech, USA), TM-β1 (BioLegend, USA), IL-15 mAbs (Invitrogen, USA), or the Erk1/2 inhibitor SCH772984 (Selleck Chemicals, USA).

### Adipogenic and Osteogenic Differentiation of MSCs

First, MSCs were grown in complete medium for adipogenic differentiation. When the cells reached 80% confluence, the medium was changed to adipogenic medium that consisted of rosiglitazone, 3-isobutyl-1-methylxanthin (IBMX), glutamine, dexamethasone and insulin (Cyagen Biosciences, Suzhou, China). Osteogenic differentiation was induced in MSCs by DMEM containing dexamethasone, ascorbic acids and β-glycerophosphate (Cyagen Biosciences, Suzhou, China). For Oil Red O staining, MSCs were fixed with 4% formaldehyde. After being washed with PBS, the cells were incubated with Oil Red O solution (Cyagen Biosciences Suzhou, China) for 30 min. The cells were then washed and examined under a microscope. For Alizarin Red S staining, the cells were incubated with Alizarin Red solution (Cyagen Biosciences, Suzhou, China) for 30 min. Images were acquired by a microscope (Supplementary Figure S1B).

### Induction of MDSCs From Bone Marrow Cells

Sterile bone marrow (BM) cells were flushed out of the tibias and femur cavities of C57BL/6 mice. Red blood cells (RBCs) were lysed by RBC lysis buffer (Invitrogen, USA). To obtain BM-derived MDSCs, BM cells were plated in dishes in RPMI 1640 medium supplemented with FBS, sodium pyruvate, MEM nonessential amino acid (NEAA) solution, 2-mercaptoethanol, streptomycin and penicillin. BM cells were induced with 40 ng/mL GM-CSF and 40 ng/ml IL-6 (PeproTech, USA) for 4 days.

### Flow Cytometry

As shown in Supplementary Figure S1C, MDSCs were sorted by a FACS Aria III flow cytometer (Becton Dickinson, CA, USA) by using mAbs against CD11b (clone: M1/70), Gr1 (clone: RB6-8C5), Ly-6G (clone: 1A8) and Ly-6C (clone: AL-21). The antibodies were purchased from BD Biosciences, United States. The sorted MDSCs were cultured in the same medium as described above at a density of 5×10^5^ cells/mL with or without IL-15 mAbs.

Splenocytes were obtained from spleens by mechanical crushing. Renal cells were obtained from the kidneys of mice by collagenase Ⅳ. Cells were examined by using mAbs against CD45 (clone: 30-F11), CD11b (clone: M1/70), Ly-6G (clone: 1A8), and Ly-6C (clone: AL-21) and DAPI (all antibodies were purchased from BD Bioscience or Biolegend Company) and analyzed by Flow JoX software.

### Animal Experiment

Male C57BL/6 mice (6 weeks old, 20–25 g) were purchased from Shanghai JieSiJie Laboratory Animal Co., Ltd. (Shanghai, China) and bred in a specific pathogen-free (SPF)-grade animal room. The mice were randomly divided into groups. 1) In the sham group, the abdomen was exposed for 30 min without renal artery clamping, and the mice were injected with 100 μL of phosphate-buffered saline (PBS); 2) in the ischemia-reperfusion injury (IRI) group, the renal pedicles of unilateral kidneys were clipped with vascular clamps for 30 min to induce ischemia, followed by injection with 100 μL of PBS; 3) in the IRI/M-MDSC supernatant group, renal IRI mice were administered M-MDSC supernatant (100 μL) via the tail vein 24 h preoperation and for seven consecutive days after the operation; and 4) in the IRI/M-MDSC supernatant/IL-15 mAb group, renal IRI mice were injected with M-MDSC supernatant plus IL-15 mAbs (100 μL) via the tail vein 24 h preoperation and for seven consecutive days after the operation. All animal procedures were authorized by the Animal Ethics Committee of Zhongshan Hospital, Fudan University.

### ELISA and Luminex Assay

IL-15 levels in cell supernatants and cytokines in murine serum were measured by a commercially available mouse IL-15 ELISA kit (Enzyme-linked Biotechnology Co., Ltd., Shanghai, China) and Bio-Plex Pro Mouse Cytokine 23-plex Assay (BioRad, USA), respectively, according to the vendor’s protocol.

### RNA Isolation and Quantification Real-Time PCR

TRIzol reagent (Thermo, CA, USA) was used to extract total RNA from each sample. cDNA was reverse-transcribed from approximately 1000 ng of total RNA by the PrimeScript™ RT reagent kit (TaKaRa, Japan). mRNA levels were quantitatively evaluated by SYBR Green-based quantitative real-time PCR in an Applied Biosystems Real-time PCR System. All primers were synthesized by Sangon Biotech (Shanghai, China). The mRNA expression levels were compared with GAPDH after normalization. The primer sequences are listed in Supplementary Table S1.

### Immunohistochemistry

Immunohistochemistry was performed on thin sections (5 μm) of formalin-fixed and paraffin-embedded tissues. The sections were deparaffinized in xylene and rehydrated in a descending ethanol series. After being heated in a microwave to repair tissue antigens, the sections were incubated with 10% normal goat serum at room temperature for 10 min to block nonspecific reactions. Monoclonal rabbit anti-CD3 (1:200; Abcam, UK) primary antibody was added and incubated at 4°C overnight, followed by the application of biotin-labeled goat anti-Ms/RbIgG and streptavidin-peroxidase (UltraSensitiveTM SP IHC Kit; Maxim, China). The sections were then developed in diaminobenzidine substrate.

### Histologic Analysis

For morphologic assessments, paraffin-embedded renal tissues 28 days after IRI were sliced into 5 μm sections and then deparaffinized and rehydrated before being stained with hematoxylin and eosin (H&E), Sirius red, and Masson trichrome and analyzed by immunohistochemistry. H&E staining was assessed to evaluate the tubular injury score by calculating the percentage of tubules that displayed tubular dilation, brush border loss, cast formation and tubular necrosis: 0, normal; 1, ≤10%; 2, 10–25%; 3, 26–50%; 4, 51–75%; 5, ≥75%. Renal fibrosis was assessed using Sirius red staining and Masson trichrome staining. The ImageJ plugin (https://imagej.nih. gov) was used to determine the positive area of Masson trichrome (blue), Sirius red (red) and immunohistochemical staining (brown). All histologic analyses were performed by two independent researchers who were blinded to the groupings.

### Immunofluorescence Analysis

Bone marrow MSCs were examined for α-SMA expression by immunofluorescence. After fixation, anti-α-SMA antibodies (1:1000, Abcam, UK) were added and incubated at 4°C overnight. Subsequently, the cells were incubated with Alexa Fluor®488 donkey anti-mouse IgG (1:400, Life Technologies, USA) at room temperature for 1 h. The nuclei were stained with DAPI (Sigma, USA). Images were obtained by a fluorescence microscope (Olympus, Tokyo, Japan).

Renal resident MSCs (RRMSCs) were examined by immunofluorescence staining for Sca-1 and α-SMA. After deparaffinization and rehydration, anti-Sca-1 (1:100, Abcam, UK) and anti-α-SMA (1:1000, Abcam, UK) antibodies were added and incubated at 4°C overnight. Subsequently, the renal sections were incubated with Alexa Fluor®594 donkey anti-mouse lgG (1:400, Life Technologies, USA) and Alexa Fluor®488 donkey anti-rabbit lgG (1:400, Life Technologies, USA) at room temperature for 1 h. Renal resident MSCs (RRMSCs) were also examined by immunofluorescence staining for Sca-1 and Ras, Sca-1 and p-Erk1/2, and Sca-1 and p-Smad3. After deparaffinization and rehydration, anti-Ras (1:800, Cell Signaling Technology, USA), anti-p-Erk1/2 (1:800, Cell Signaling Technology, USA) or anti-p-Smad3 (1:500, Abcam, UK) antibodies were added and incubated at 4°C overnight. Subsequently, the renal sections were incubated with goat anti-rabbit IgG (HRP, 1:4000, Abcam, UK) at room temperature for 1 h. CY5 (1:200, Abcam, UK) was then used, and the sections were incubated for 10 min. After another rehydration step, anti-Sca-1 (1:100, Abcam, UK) antibodies were added and incubated at 4°C overnight. Then, the renal sections were incubated with Alexa Fluor^®^488 donkey anti-rabbit lgG (1:400, Life Technologies, USA) at room temperature for 1 h. The nuclei were stained with DAPI (Sigma, USA). Images were obtained by a fluorescence microscope (Olympus, Tokyo, Japan). The area of co-stained Sca-1^+^α-SMA^+^ renal resident MSCs was quantified by using the colocalization finder plugin of ImageJ.

### Western Blotting

Twenty micrograms of protein from MSC homogenates was separated on 15% or 10% polyacrylamide denaturing gels and electroblotted onto polyvinylidene fluoride membranes. The primary antibodies used were α-SMA (1:1000, Abcam, USA), col1α1 (1:1000, Abcam, USA), Ras (1:1000, Cell Signaling Technology, USA), p-Erk1/2 (1:1000, Cell Signaling Technology, USA), Erk1/2 (1:1000, Cell Signaling Technology, USA), p-Smad2/3 (1:1000, Cell Signaling Technology, USA), and Smad2/3 (1:1000, Cell Signaling Technology, USA). The results were normalized to tubulin (1:1000 dilution, Abcam).

### RNA Sequencing

RNA was isolated from control MSCs, TGF-β1-induced MSCs, TGF-β1-induced MSCs with M-MDSC supernatant and TGF-β1-induced MSCs with G-MDSC supernatant with TRIzol reagent (Thermo, CA, USA). Paired-end sequencing (PE150, 2 × 150 bp) was performed on an Illumina NovaSeq™ 6,000 (LC-Bio Technology CO., Ltd., Hangzhou, China) according to the manufacturer’s recommended protocol. The read quality was also verified using Fastp software (https://github.com/OpenGene/fastp). Reads were aligned to the reference genome of Mus musculus (GRCm38) by HISAT2 (https://ccb.jhu.edu/software/hisat2) and assembled by StringTie (https://ccb.jhu.edu/software/stringtie). Subsequently, all transcriptomes were merged using gffcompare (https://github.com/gpertea/gffcompare/). The expression level of mRNAs was calculated by FPKM (FPKM = [total_exon_fragments/mapped_reads (millions) × exon_length (kB)]). The differentially expressed mRNAs were chosen (fold change >2 or fold change <0.5, *p* value <0.05) by the R package edgeR (https://bioconductor.org/packages/release/bioc/html/edgeR.html).

### Statistical Analysis

Images of flow charts were created with biorender.com. The data were analyzed using GraphPad Prism eight software or R language. Packages such as Limma, Annotation, ggplot2, and enrichGo were used to analyze the RNA sequencing and array results. Quantitative variables were analyzed by two-tailed independent *t* test (between two groups) and are expressed as the means ± standard deviation (S.D.). *p* < 0.05 was considered statistically significant.

## Results

### MDSC Supernatant Inhibits TGF-β1-Induced Myofibroblastic Differentiation in MSCs

First, we identified BM-MSCs by specific MSC surface markers through flow cytometry (Supplementary Figure S1A). These cells were positive for CD29, CD44, and Sca-1 but negative for CD117 and CD31. Their adipogenic and osteogenic potential were evaluated by oil red O and Alizarin red S staining, respectively (Supplementary Figure S1B).

**FIGURE 1:**
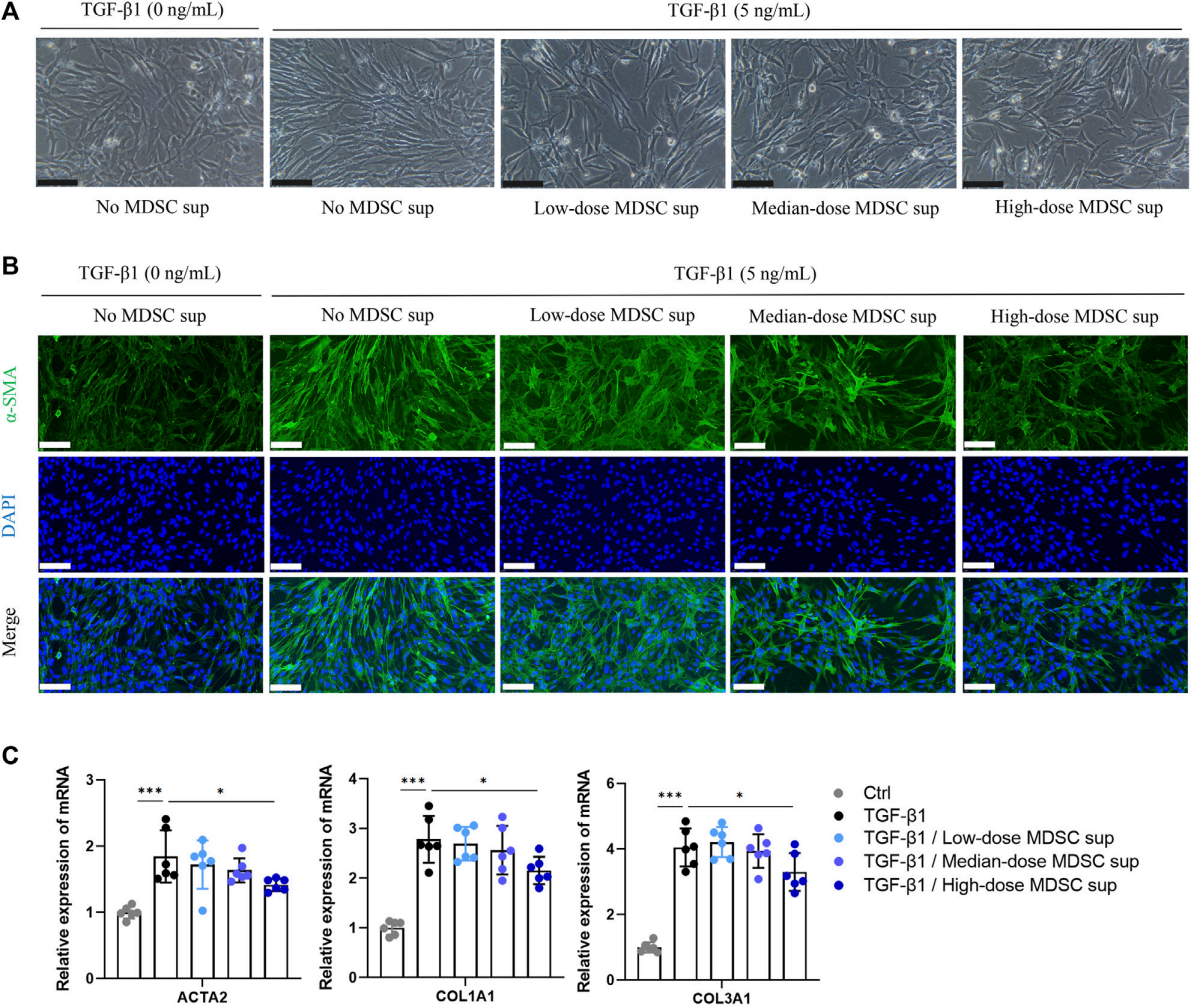
MDSC supernatant inhibits the TGF-β1-induced myofibroblastic differentiation of MSCs. **(A)** BM-derived CD11b^+^Gr^+^ MDSCs were obtained by flow cytometry and cultured in normal medium for 24 h at a density of 5×10^4^ cells/mL (low dose), 1×10^5^ cells/mL (medium dose), and 5×10^5^ cells/mL (high dose). Then, the culture supernatants were collected. MSCs were cultured with different doses of MDSC supernatant containing TGF-β1 (5 ng/mL) for 24 h, and cell morphology was observed under a microscope. Scale bar: 100 μm. **(B)** Immunofluorescence staining of α-SMA in MSCs. Scale bar: 100 μm. **(C)** mRNA expression of ACTA2, COL1A1 and COL3A1 in MSCs was normalized to GAPDH expression and analyzed by qPCR. Values were expressed as mean ± standard deviation (mean ± SD). *p < 0.05; **p < 0.01; ***p < 0.001.

To ascertain the effect of MDSCs on TGF-β1-induced myofibroblastic differentiation in MSCs *in vitro*, BM-derived CD11b^+^Gr^+^ MDSCs were obtained (Supplementary Figure S1C) and cultured in medium for 24 h at a density of 5×10^4^ cells/mL (low dose), 1×10^5^ cells/mL (medium dose), and 5×10^5^ cells/mL (high dose). Then, the culture supernatants were collected. The myofibroblastic differentiation of MSCs was evaluated in the presence of different doses of MDSC supernatant (MDSC sup). We found that TGF-β1 (5 ng/mL) triggered a myofibroblastic differentiation process that was characterized by an alteration in cell morphology from the characteristic organized spindle-shaped appearance to a disorganized elongated fibroblast-like phenotype, and this effect was attenuated by treatment with high-dose MDSC supernatant (Figure 1A). Immunofluorescence staining showed that the expression of α-SMA, a marker of myofibroblasts, was upregulated by TGF-β1 induction (Figure 1B). These results were consistent with a significant increase in the levels of the myofibroblastic differentiation-related genes ACTA2, COL1A1 and COL1A3 (Figure 1C). In contrast, MSCs induced by TGF-β1 that were treated with high-dose MDSC supernatant displayed dramatically decreased levels of ACTA2, COL1A1 and COL1A3 (Figures 1B,C). These results demonstrate that MDSC supernatant effectively inhibits TGF-β1-induced myofibroblastic differentiation in MSCs.

### M-MDSC Supernatant Inhibits TGF-β1-Induced Myofibroblastic Differentiation in MSCs

**FIGURE 2:**
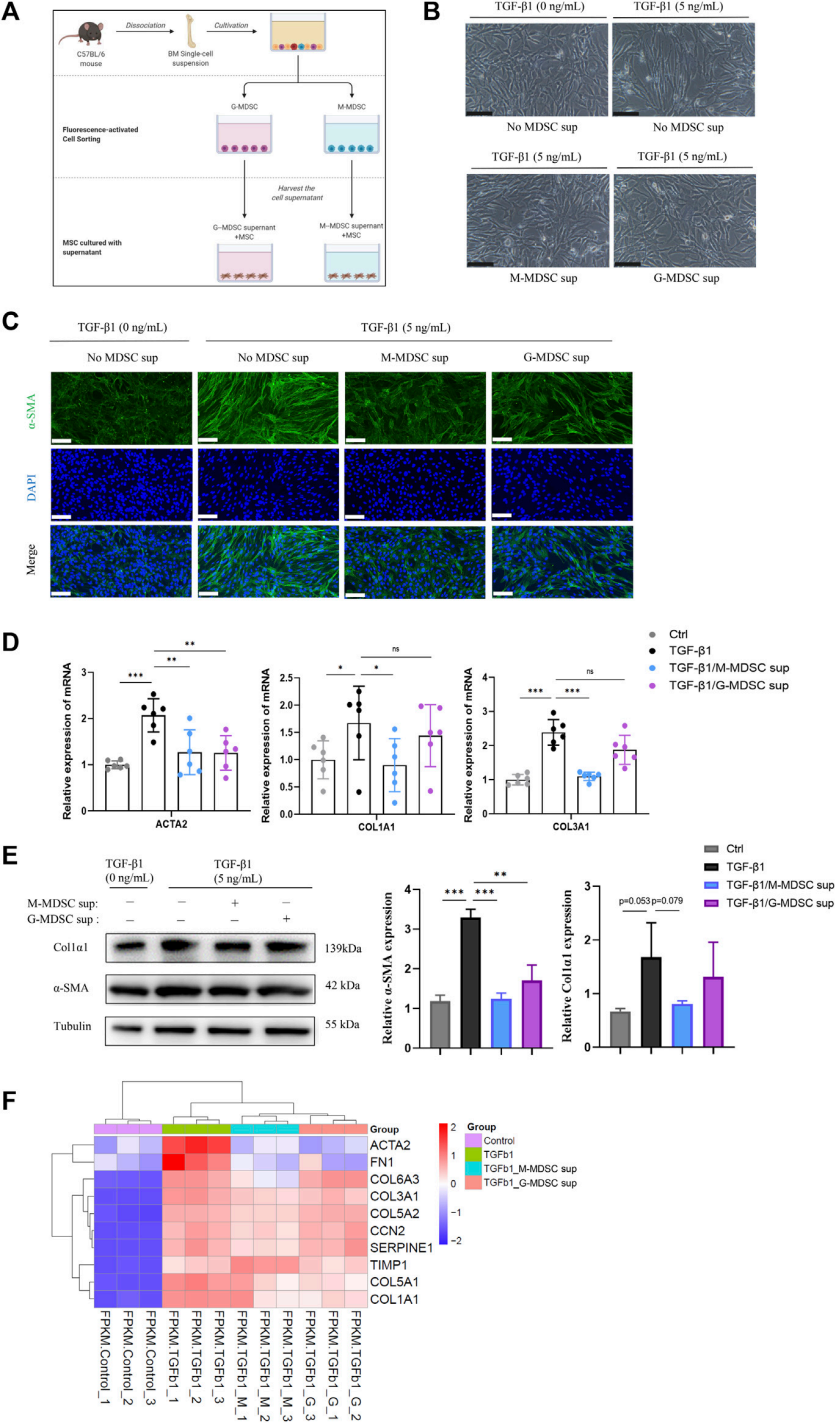
M-MDSC supernatant inhibits the TGF-β1-induced myofibroblastic differentiation of MSCs. **(A)** Bone marrow CD11b^+^Ly6G^−^Ly6C^high^ M-MDSCs and CD11b^+^Ly6G^+^Ly6C^low^ G-MDSCs were sorted by flow cytometry, and the culture supernatants were collected after another 24 h of culture in normal medium. The myofibroblastic differentiation of MSCs was evaluated in the presence of G-MDSC supernatant or M-MDSC supernatant containing TGF-β1 (5 ng/mL) for 24 h. **(B)** Morphology of MSCs treated with TGF-β1, TGF-β1/M-MDSC supernatant or TGF-β1/G-MDSC supernatant. Scale bar: 100 μm. **(C)** Immunofluorescence staining of α-SMA in MSCs. Scale bar: 100 μm. **(D)** mRNA expression of ACTA2, COL1A1 and COL3A1 in MSCs was normalized to GAPDH expression and analyzed by qPCR. Values were expressed as mean ± standard deviation (mean ± SD). **(E)** Representative western blot and average data for α-SMA and col1α1 in mesenchymal stem cells of each experimental group, normalized to expression of tubulin. Values were expressed as mean ± standard deviation (mean ± SD). **(F)** A heatmap of ACTA2, COL1A1, COL3A1, COL5A1, COL5A2, COL6A3, FN1, SERPINE1, CCN2 and TIMP1 expression in each sample. *p < 0.05; **p < 0.01; ***p < 0.001.

To explore the effects of MDSC subtypes on TGF-β1-induced myofibroblastic differentiation in MSCs *in vitro*, bone marrow CD11b^+^Ly6G^−^Ly6C^high^ M-MDSCs and CD11b^+^Ly6G^+^Ly6C^low^ G-MDSCs were sorted by flow cytometry (Supplementary Figure S1C), and the culture supernatants were collected. As shown in Figure 2A, the myofibroblastic differentiation of MSCs was evaluated in the presence of different MDSC supernatants (G-MDSC sup and M-MDSC sup). M-MDSC supernatant and G-MDSC supernatant both attenuated the TGF-β1-induced myofibroblastic differentiation process, which was observed by cell morphology (Figure 2B), and decreased the expression of α-SMA, as shown by immunofluorescence staining (Figure 2C). Interestingly, TGF-β1 treatment markedly increased the expression of ACTA2, COL1A1 and COL1A3, while the levels of COL1A1 and COL1A3 were downregulated in the M-MDSC supernatant-treated group but not the G-MDSC supernatant-treated group (Figure 2D). Moreover, the decreased levels of α-SMA and COL1A1 induced by MDSC supernatant treatment were also confirmed through western blot analysis (Figure 2E).

To further investigate MDSC subtype-derived supernatant-mediated inhibition of TGF-β1-induced myofibroblast differentiation in MSCs, we analyzed the mRNA expression in the four groups, including MSCs without treatment, MSCs with TGF-β1 treatment, MSCs with TGF-β1/M-MDSC supernatant treatment and MSCs with TGF-β1/G-MDSC supernatant treatment, using mRNA sequencing. We found that the mRNA expression of myofibroblastic differentiation-related genes, such as ACTA2, COL1A1, COL3A1, COL5A1, COL5A2, COL6A3, FN1, SERPINE1, CCN2 and TIMP1, was upregulated by TGF-β1. Most of these markers were reduced by MDSC supernatant treatment, and the effect was more robust in the M-MDSC supernatant-treated group than in the G-MDSC supernatant-treated group (Figure 2F **)**. We therefore focused on M-MDSCs, which may be more relevant to the inhibition of TGF-β1-induced myofibroblast differentiation. Taken together, these results suggested that M-MDSC supernatant showed more potent abilities to impair the TGF-β1-induced myofibroblastic differentiation of MSCs.

### M-MDSC-Derived IL-15 Plays a Critical Role in Inhibiting Myofibroblastic Differentiation in MSCs

**FIGURE 3:**
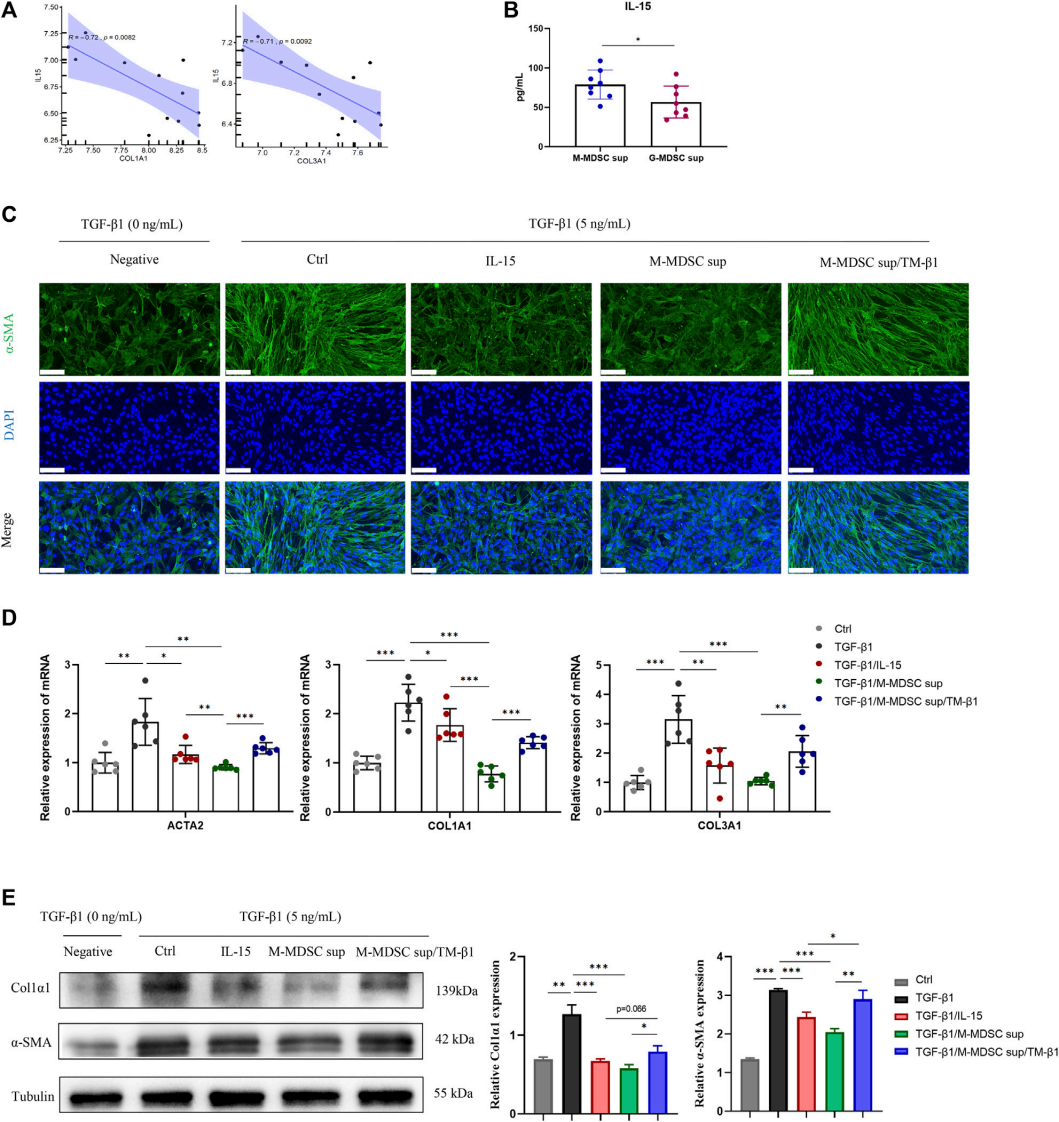
M-MDSC-derived IL-15 plays a critical role in the suppression of myofibroblastic differentiation. **(A)** The expression of COL1A1 and COL3A1 was inversely related to IL-15 expression in the published GSE83610 dataset. **(B)** The level of IL-15 in M-MDSC and G-MDSC supernatants was measured by ELISA. **(C)** MSCs were cultured with TGF-β1 (5 ng/mL) for 24 h with or without IL-15 (1 ng/mL), M-MDSC supernatant and TM-β1 (10 ng/mL). α-SMA in MSCs was measured by immunofluorescence staining. Scale bar: 100 μm. **(D)** mRNA expression of ACTA2, COL1A1 and COL3A1 in MSCs was normalized to GAPDH expression. Values were expressed as mean ± standard deviation (mean ± SD). **(E)** Representative western blot and average data for α-SMA and col1α1 in mesenchymal stem cells of each experimental group, normalized to expression of tubulin. Values were expressed as mean ± standard deviation (mean ± SD). *p < 0.05; **p < 0.01; ***p < 0.001.

Given that MDSCs produce various types of cytokines that play important roles in the immune system, we focused on secreted cytokines in M-MDSC supernatant. Since intrarenal IL-15 is strongly decreased in several experimental murine nephropathies and human renal dysfunction, we investigated the IL-15 expression profile of the renal medulla of lithium-induced renal fibrosis by bioinformatics based on Gene Expression Omnibus (GEO) datasets. Myofibroblast-related markers, including COL1A1 and COL3A1, were inversely related to IL-15 expression in published GSE83610 dataset (Figure 3A). ELISA was performed to show that the level of IL-15 in M-MDSC supernatant was higher than that in G-MDSC supernatant (Figure 3B). We next investigated whether IL-15 could account for the anti-myofibroblastic effect of M-MDSC supernatant. Immunofluorescence analysis revealed that TGF-β1 treatment strongly increased α-SMA expression in MSCs, and this production was markedly suppressed by IL-15 (1 ng/mL) or M-MDSC supernatant treatment. These inhibitory effects could be reversed by TM-β1 (10 ng/mL), a specific Ab targeting IL-15 receptor β (Figure 3C). The expression of myofibroblastic markers was altered, as shown by qPCR (Figure 3D) and western blot analysis (Figure 3E). Collectively, our results suggest that IL-15 is partially responsible for the inhibitory effect of M-MDSC culture supernatant. Thus, IL-15, which is secreted by M-MDSCs, can potently inhibit the TGF-β1-induced myofibroblastic differentiation of MSCs.

### The Effects of M-MDSC-Derived IL-15 on Myofibroblastic Differentiation of MSCs are Mediated by Ras-Erk1/2-Smad2/3 Signaling

**FIGURE 4:**
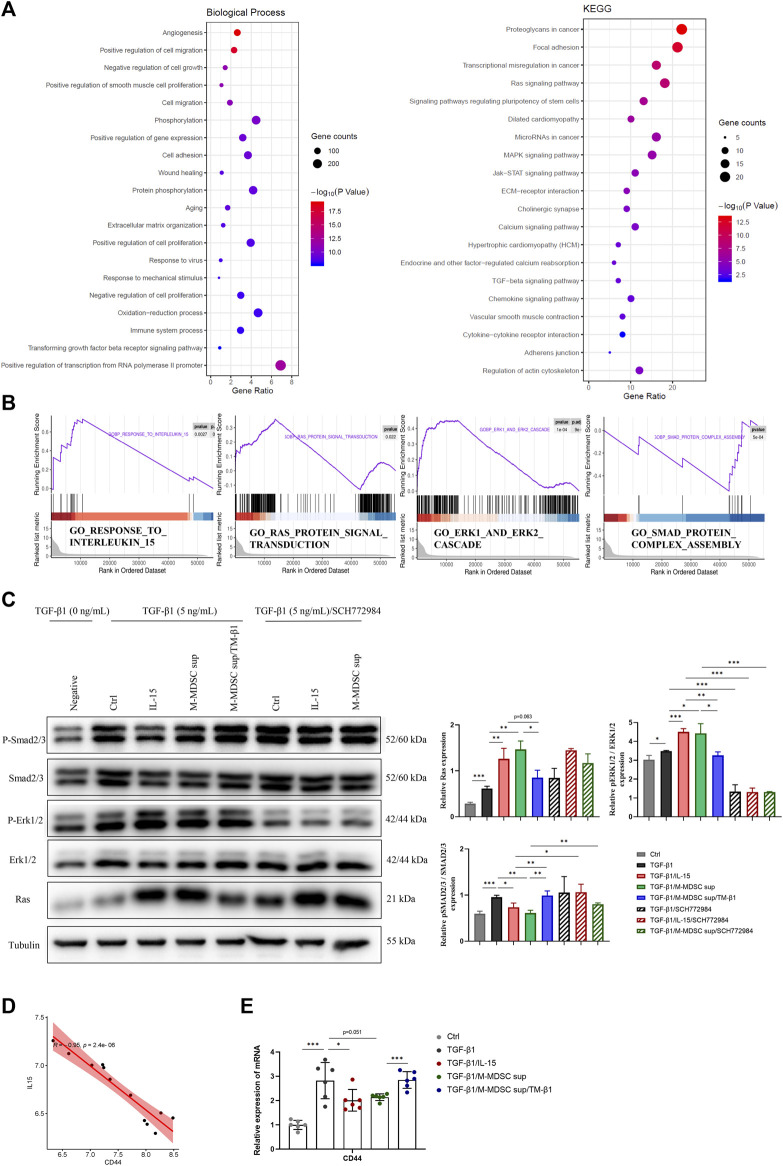
The effects of M-MDSC supernatant on the myofibroblastic differentiation of MSCs are mediated by IL-15-Ras-Erk1/2-Smad2/3 signaling. **(A)** Based on differentially expressed genes (DEGs) between MSCs treated with TGF-β1 and MSCs treated with TGF-β1/M-MDSC supernatant, the enrichment items were analyzed by Gene Ontology (GO) functional annotation and Kyoto Encyclopedia of Genes and Genomes (KEGG) pathway analysis. **(B)** Gene set enrichment analysis (GSEA) analysis was performed to screen significantly enriched pathways. **(C)** MSCs were cultured with TGF-β1 (5 ng/mL) for 24 h with or without IL-15 (1 ng/mL), M-MDSC supernatant, TM-β1 (10 ng/mL) and SCH772984 (10 μM). The protein expression levels of Ras, p-Erk1/2, Erk1/2, p-Smad2/3 and Smad2/3 were detected by western blotting. Values were expressed as mean ± standard deviation (mean ± SD). **(D)** The expression of CD44 was inversely related to IL-15 expression in the published GSE83610 dataset. **(E)** MSCs were cultured with TGF-β1 (5 ng/mL) for 24 h with or without IL-15 (1 ng/mL), M-MDSC supernatant and TM-β1 (10 ng/mL). The mRNA expression of CD44 in MSCs was normalized to GAPDH expression. Values were expressed as mean ± standard deviation (mean ± SD). *p < 0.05; **p < 0.01; ***p < 0.001.

To investigate the mechanism mediating the effects of M-MDSCs on TGF-β1-induced myofibroblastic differentiation in MSCs, Gene Ontology (GO) functional annotation and Kyoto Encyclopedia of Genes and Genomes (KEGG) pathway analysis based on mRNA sequencing data were performed (Figure 4A). TGF-β1/M-MDSC supernatant treatment of MSCs was associated with angiogenesis, the regulation of cell migration, cell proliferation, gene expression, cell adhesion and wound healing based on GO analysis. According to KEGG pathway analysis, MSCs treated with TGF-β1/M-MDSC supernatant were associated with the Ras signaling pathway, pluripotency of stem cells, MAPK signaling pathway, JAK-STAT signaling pathway and TGF-β1 signaling pathway. Furthermore, gene set enrichment analysis (GSEA) revealed the hallmarks of M-MDSC treatment, including the response to interleukin 15, Ras protein signal transduction, Smad protein complex assembly and the Erk1/2 cascade (Figure 4B). We thus assessed whether the Ras-Erk1/2-Smad2/3 pathway was involved in M-MDSC supernatant-mediated inhibition of myofibroblastic differentiation in MSCs. Western blotting revealed that M-MDSC supernatant and IL-15 induced a marked increase in Ras and p-Erk1/2 expression and a decrease in p-Smad2/3 in MSCs treated with TGF-β1. This effect was blocked when the cells were incubated with TM-β1. Moreover, an Erk1/2 inhibitor abolished the downregulation of p-Smad2/3 induced by IL-15 or M-MDSC supernatant (Figure 4C). These results suggest that M-MDSC supernatant exerts inhibitory effects through IL-15/Ras/Erk/Smad signaling.

According to the results of the GO analysis, we further studied the expression of CD44, an adhesion and migration marker on the cell membrane of MSCs. By analyzing the published GSE83610 dataset, CD44 was shown to be inversely related to IL-15 expression in the rat renal medulla of lithium-induced renal fibrosis (Figure 4D). M-MDSC supernatant downregulated CD44 expression in MSCs via IL-15, as determined by qPCR (Figure 4E).

### Treatment With M-MDSC Supernatant Ameliorates Renal Fibrosis and Myofibroblastic Differentiation of RRMSCs

**FIGURE 5:**
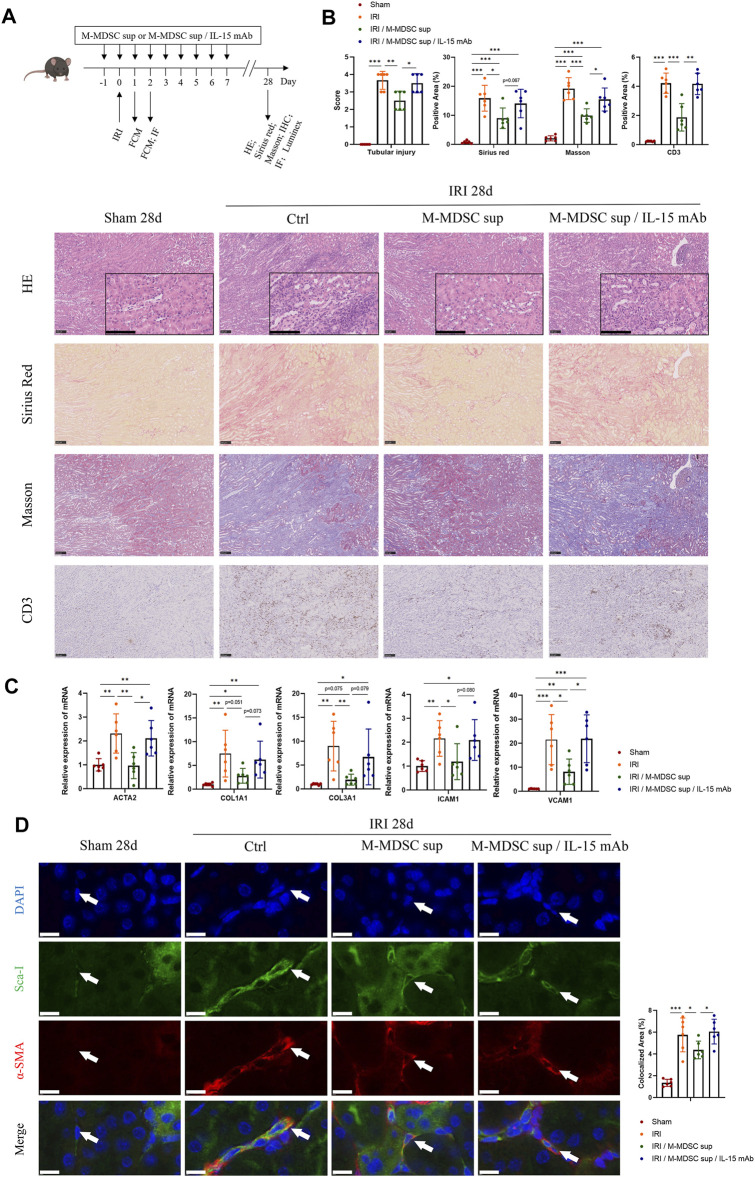
Treatment with M-MDSC supernatant ameliorates renal fibrosis and myofibroblastic differentiation in RRMSCs. **(A)** M-MDSC supernatant or M-MDSC supernatant/IL-15 mAb was administered to C57BL/6 mice 1 day prior to the IRI operation and for seven consecutive days after the IRI operation. The bone marrow and spleen were harvested on Days 1 and 2, and kidneys were harvested on Days 1, 2 and 28 after the operation. **(B)** On Day 28 after the operation, tubular injury was measured by H&E, while renal fibrosis was measured by Sirius Red and Masson trichrome staining. The expression of CD3 was measured by immunohistochemistry. Scale bar: 100 μm. **(C)** On Day 28 after the operation, the mRNA expression of ACTA2, COL1A1, COL3A1, ICAM1 and VCAM1 in renal tissues was normalized to GAPDH expression. Values were expressed as mean ± standard deviation (mean ± SD). **(D)** Immunofluorescence costaining of Sca-1/α-SMA in the kidney on Day 28 after the operation. Average data for area of co-stained Sca-1^+^α-SMA^+^ renal resident MSCs were quantified. Values were expressed as mean ± standard deviation (mean ± SD). Scale bar: 10 μm. IHC: Immunohistochemistry; FCM: Flow cytometry; IF: Immunofluorescence staining. *p < 0.05; **p < 0.01; ***p < 0.001.

As shown in Figure 5A, to further verify the effect of M-MDSC supernatant treatment on myofibroblast differentiation in tissue resident MSCs in the context of renal fibrosis, mice were administered M-MDSC supernatant following renal unilateral ischemia-reperfusion injury (IRI), with or without IL-15 blockade by IL-15 mAbs (10 μg/mL). Twenty-eight days after unilateral renal IRI, we found that transfusion of M-MDSC supernatant significantly reduced damage to renal tubules and the infiltration of CD3^+^ T cells, which was reversed by IL-15 mAb treatment, as assessed by H&E staining and immunohistochemistry. Sirius red and Masson staining showed that M-MDSC supernatant downregulated IRI-induced renal fibrosis, which could be reversed by IL-15 mAb treatment (Figure 5B). Renal mRNA (Figure 5C) levels of fibrotic markers (ACTA2, COL1A1 and COL3A1) and adhesion molecules (ICAM1 and VCAM1) were decreased following M-MDSC supernatant treatment but rebounded after IL-15 mAb treatment. Dual immunofluorescence analyses showed the localization of some RRMSCs coexpressing the stem cell marker Sca-1 and the myofibroblast marker α-SMA, which was consistent with the concept that the myofibroblastic differentiation of RRMSCs increased during renal fibrosis. Moreover, the colocalization of Sca-1 and α-SMA was reduced after M-MDSC supernatant transfer. This effect was counteracted by IL-15 mAb treatment (Figure 5D). These findings suggest that M-MDSC supernatant treatment following IRI attenuates renal fibrosis and myofibroblast differentiation in RRMSCs, probably through IL-15.

### M-MDSC Supernatant Attenuated Systemic Inflammation Associated With Renal Fibrosis by Promoting M-MDSCs in the BM, Spleen and Kidney via IL-15

**FIGURE 6:**
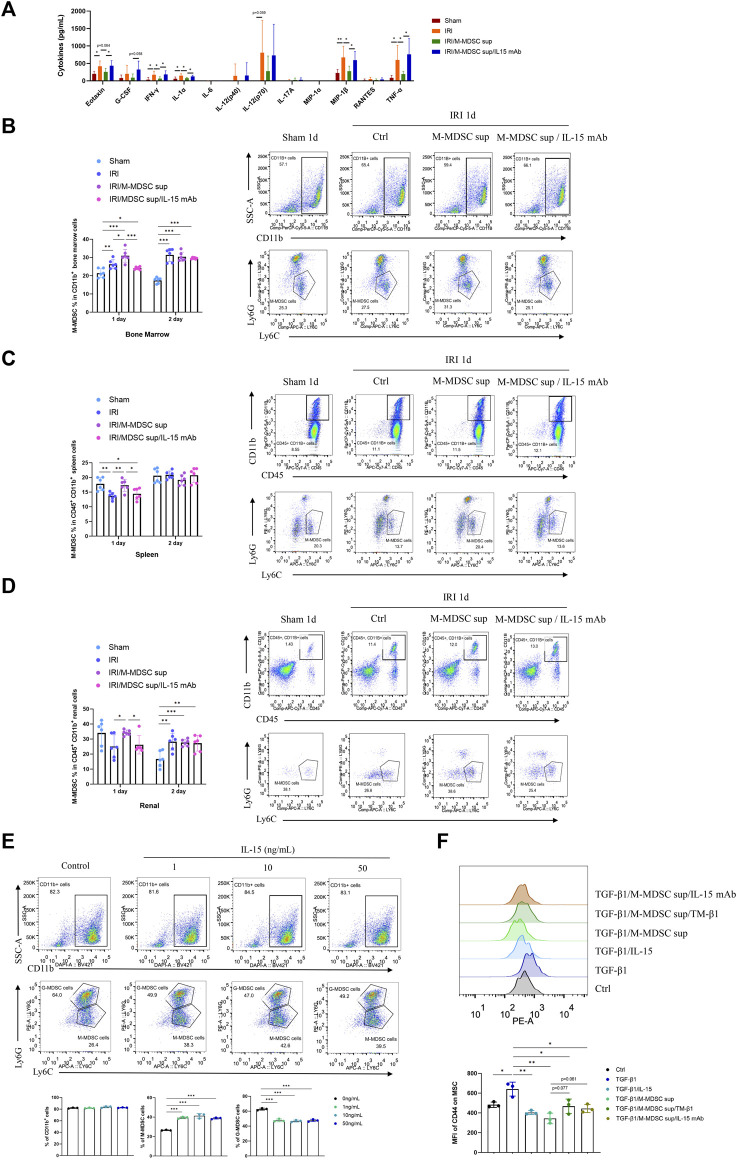
M-MDSC supernatant attenuated systemic inflammation in renal fibrosis by promoting M-MDSCs in the BM, spleen and kidney via IL-15. **(A)** Inflammatory cytokines in the serum of the murine renal IRI-induced fibrosis model were determined by Luminex analysis on Day 28 after the operation. **(B)** Detection of the proportion of CD11b^+^Ly6G^−^Ly6C^high^ M-MDSCs in the bone marrow by flow cytometry on Days 1 and 2 after the operation. **(C)** Detection of the proportion of CD11b^+^Ly6G^−^Ly6C^high^ M-MDSCs in the spleen by flow cytometry on Days 1 and 2 after the operation. **(D)** Detection of the proportion of CD11b^+^Ly6G^−^Ly6C^high^ M-MDSCs in the kidney by flow cytometry on Days 1 and 2 after the operation. **(E)** Detection of CD11b^+^ myeloid cells, CD11b^+^Ly6G^−^Ly6C^high^ M-MDSCs, and CD11b^+^Ly6G^+^Ly6C^low^ G-MDSCs in the bone marrow by flow cytometry after induction with IL-15 for 4 days. **(F)** Detection of the median fluorescence intensity (MFI) of CD44 adhesion molecule expression on the surface of MSCs after induction with TGF-β1 (5 ng/mL) for 24 h, with or without IL-15 (1 ng/mL), M-MDSC supernatant, IL-15 mAb (10 μg/mL) and TM-β1 (10 ng/mL) for 2 days. *p < 0.05; **p < 0.01; ***p < 0.001.

Renal fibrosis is accompanied by systemic inflammation. Thus, we evaluated whether M-MDSC supernatant affected systemic inflammation in renal fibrosis. Luminex assay was used to examine cytokines in the serum of the murine renal IRI-induced fibrosis model. We found that M-MDSC supernatant attenuated the systemic inflammation associated with renal fibrosis via IL-15 (Figure 6A). IL-15 could regulate the proliferation of T cells and NK cells. However, whether IL-15 participates in regulating different MDSCs during renal fibrosis needs further study. Thus, we performed flow cytometry to explore the effect of MDSC supernatant or MDSC supernatant plus IL-15 mAbs on M-MDSC proliferation in the bone marrow, spleen and kidney at 24 and 48 h after renal IRI. M-MDSC supernatant increased the infiltration of M-MDSCs in the bone marrow (Figure 6B), spleen (Figure 6C) and kidney (Figure 6D) at 24 h after IRI, which was abolished by IL-15 mAb treatment. Moreover, we observed that IL-15 treatment increased the number of M-MDSCs among bone marrow cells *ex vivo* (Figure 6E). These data demonstrated that M-MDSC supernatant treatment increased the infiltration of M-MDSCs after IRI, possibly through IL-15.

Moreover, TGF-β1 treatment upregulated CD44 on the cell membrane of MSCs, and this effect was suppressed by IL-15 or M-MDSC supernatant treatment. These inhibitory effects could be reversed by IL-15 mAb and TM-β1 (Figure 6F), suggesting that M-MDSC supernatant may reduce the adhesion and migration of MSCs via IL-15.

### The Effects of M-MDSCs on the Myofibroblastic Differentiation of Renal Resident MSCs are Mediated by IL-15-Ras-Erk1/2-Smad2/3 Signaling

**FIGURE 7:**
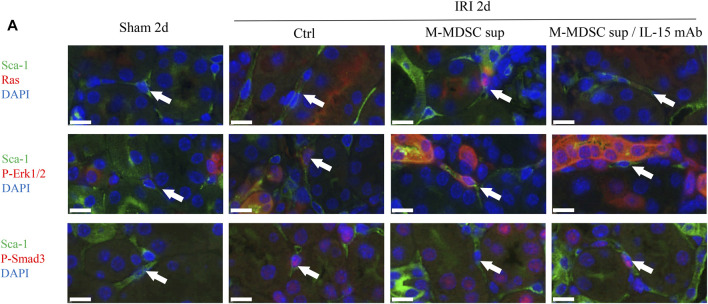
The effects of M-MDSCs on the myofibroblastic differentiation of renal resident MSCs are mediated by IL-15-Ras-Erk1/2-Smad2/3 signaling. **(A)** Immunofluorescence costaining of Sca-1/Ras, Sca-1/p-Erk1/2 and Sca-1/p-Smad3 in the kidney on Day 2 after the operation. Scale bar: 10 μm *p < 0.05; **p < 0.01; ***p < 0.001.

We examined whether the IL-15-Ras-Erk1/2-Smad2/3 pathway was involved in M-MDSC supernatant-mediated inhibition of the myofibroblastic differentiation of RRMSCs. At 2 days after surgery, dual immunofluorescence analysis demonstrated that M-MDSC supernatant and IL-15 induced marked upregulation of Ras and p-Erk1/2 expression and downregulation of p-Smad3 in RRMSCs expressing Sca-1. These effects were blocked by IL-15 mAbs (Figure 7A).

## Discussion

**FIGURE 8:**
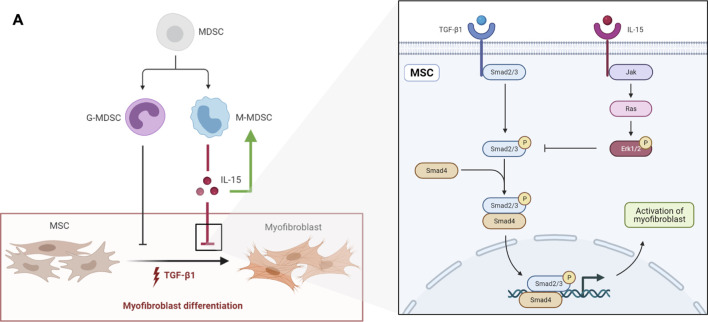
**(A)** schematic diagram depicting the effects of MDSCs on the myofibroblastic differentiation of MSCs. G-MDSCs and M-MDSCs both have inhibitory effects on the myofibroblastic differentiation of MSCs. M-MDSCs exert inhibitory effects through IL-15/Ras/Erk/Smad signaling. Additionally, M-MDSC supernatant promoted the proliferation of M-MDSCs sup via IL-15.

Myofibroblast differentiation is a central event in tissue fibrosis, including renal fibrosis. Recent research has revealed that myofibroblasts are derived from circulating BM-MSCs and resident MSC-like cells. Therefore, it is crucial to explore a way to inhibit the myofibroblastic differentiation of MSCs during fibrosis. MDSCs, including M-MDSCs and G-MDSCs, have been shown to play an important role in fibrosis. However, the effects of MDSCs vary under different pathophysiological conditions. In this study, we demonstrated that M-MDSCs inhibited the myofibroblastic differentiation of MSCs through IL-15 secretion. Additionally, M-MDSC supernatant further promoted the proliferation of M-MDSC supernatant via IL-15 (Figure 8).

First, we showed that both G-MDSC and M-MDSC supernatants could inhibit TGF-β1-induced myofibroblastic differentiation in MSCs to different extents. It seemed that M-MDSC supernatant had a stronger inhibitory effect on the expression of TGF-β1-induced myofibroblastic markers than G-MDSC supernatant. We therefore focused on M-MDSCs. As MDSCs play a role in immunosuppression by producing various cytokines and exosomes, we aimed to determine which cytokine might be involved in inhibiting myofibroblastic differentiation in MSCs.

Previous research showed that IL-15 expression was decreased in transplants with renal dysfunction and human inflammatory nephropathies, including focal segmental glomerulosclerosis, diabetic nephropathy, IgA nephropathy, hypertensive nephropathy, minimal change disease, membranous glomerulonephritis and thin basement membrane disease (Devocelle et al., 2019). Based on these findings, we examined IL-15 expression in the supernatants of M-MDSCs and G-MDSCs. As expected, IL-15 levels in M-MDSC supernatant were higher than those in G-MDSC supernatant. We next investigated whether IL-15 accounted for the anti-myofibroblastic effect of M-MDSC supernatant. The results showed that the TGF-β1-induced myofibroblastic differentiation potential of MSCs was partially inhibited by IL-15 treatment. The suppressive effect of M-MDSC supernatant on TGF-β1-induced myofibroblastic differentiation in MSCs was relatively abolished by TM-β1, an antibody against IL-15 receptor β. However, there must be other cytokines in M-MDSC supernatant that impair myofibroblastic differentiation. In addition, some studies suggest that the membrane-bound form but not the soluble form of IL-15 is the dominant physiological isoform of the cytokine (Fiore et al., 2020). These findings may explain why the inhibitory effect of exogenous IL-15 is less than that of M-MDSC supernatant. The cytokines in M-MDSC supernatant need to be further studied.

Recent data in other cell models have shown that IL-15 does not inhibit the initial steps of the TGF-β1-Smad2/3 signaling pathway (Devocelle et al., 2019). Based on our KEGG pathway and GSEA results, we assessed whether the Ras-Erk1/2-Smad2/3 pathway was involved in M-MDSC supernatant- or IL-15-mediated inhibition of myofibroblastic differentiation in MSCs. Indeed, M-MDSC supernatant and IL-15 increased Ras and p-Erk1/2 expression and decreased p-Smad2/3 in MSCs treated with TGF-β1, and this effect was reversed by TM-β1. Erk1/2 inhibitors abolished the downregulation of p-Smad2/3 induced by IL-15 or M-MDSC supernatant. These results suggested that M-MDSC supernatant and IL-15 inhibited Smad2/3 signaling by upregulating Ras-Erk1/2. Further verification with other specific signaling pathway inhibitors is needed in the future.

Lung-resident mesenchymal stem cells (LR-MSCs) have been reported to be vital regulators of fibrosis, and Cao et al. (2020) found that inhibiting the Shh-Wnt signaling cascade prevented LR-MSC transformation into myofibroblasts in a mouse pulmonary fibrotic model by immunofluorescence costaining of α-SMA and Sca-1. Similarly, accumulating evidence indicates that renal tissue contains a population of renal resident MSCs (RRMSCs) that are precursors of myofibroblasts, which play an important role in renal fibrosis. In addition, RRMSC populations have been proven to support strong congruence with bone marrow MSCs. Thus, to verify the effect of M-MDSC supernatant treatment *in vivo*, we administered M-MDSC supernatant following renal unilateral IRI to observe myofibroblast differentiation in renal resident MSCs (RRMSCs) at 28 days after IRI by costaining for a marker of myofibroblasts (α-SMA) and a marker of RRMSCs. Jiang et al. (2015) isolated renal Nestin^+^ cells that fulfilled all of the criteria of mesenchymal stem cells. Kramann et al. (2015) showed that Gli1 marks a network of perivascular MSC-like cells that generate myofibroblasts and play a central role in kidney fibrosis. Pelekanos et al. (2012) sorted CD45^−^CD31^−^Sca-1^+^CD24^lo^ kidney cells from C57BL/6 mice, which were verified to display an MSC-like phenotype. We isolated CD45^−^CD11b^−^CD31^−^Sca-1^+^ renal cells and examined their adipogenic and osteogenic differentiation abilities (Supplementary Figure S2). Therefore, we chose Sca-1 as the marker of RRMSCs in this study. The results showed that M-MDSC supernatant ameliorated renal fibrosis. Importantly, we found that M-MDSC supernatant hindered myofibroblastic differentiation in RRMSCs after IRI by costaining α-SMA and Sca-1. These effects of M-MDSC supernatant could be abolished by IL-15 mAbs. Corresponding changes in the Ras-Erk1/2-Smad2/3 pathway in RRMSCs were observed by immunofluorescence costaining and were consistent with the *in vitro* results.

Previous studies have reported that IL-15 is associated with the induction of various immune cells. Guo et al. (2020) found that IL-15/IL-15Ra complex treatment diminished MDSCs in murine breast tumors. We next investigated whether IL-15 participated in regulating MDSCs during renal fibrosis. Interestingly, our data demonstrated that M-MDSC supernatant treatment increased bone marrow, spleen and renal infiltration of M-MDSCs at 24 h after IRI, and this effect could be reversed by IL-15 mAbs. In addition, IL-15 treatment upregulated M-MDSCs *in vitro*. The underlying mechanisms still need to be further investigated.

CD44 is one of the most abundant receptors on MSCs and plays a major role in cell adhesion and migration (Ouhtit et al., 2020). Sierra-Parraga et al. (2020) showed that MSCs upregulated their migration and adhesion to injured endothelial cells, which was mediated by CD44 on the MSC membrane. In our study, M-MDSC supernatant and IL-15 regulated CD44 expression on the cell membrane of TGF-β1-induced MSCs, which suggested that M-MDSC supernatant and IL-15 may be involved in reducing the adhesion and migration of MSCs during renal fibrosis.

In summary, this study explored the interaction between MDSCs and MSCs and found that MDSC-derived supernatants could attenuate myofibroblastic differentiation in MSCs. The inhibitory effect was found to be partially mediated by IL-15-Ras-Erk1/2-Smad2/3 signaling. Treatment with M-MDSC supernatant ameliorated renal fibrosis and myofibroblastic differentiation in RRMSCs via IL-15, which may have implications in ameliorating renal fibrosis.

## Data Availability

Transcriptome sequence data have been deposited in NCBI GEO (GEO accession number GSE193592).

## Supplementary Material

Supplementary Figure S1 Isolation of MDSCs and identification of MSCs. **(A)** Classic surface markers of BMSCs were detected by flow cytometry. Oil red O staining and Alizarin Red staining **(B)** of bone marrow MSCs after the induction of adipogenic and osteogenic differentiation. **(C)** Isolation of MDSCs by fluorescence-activated cell sorting (FACS).

Supplementary Figure S2 Isolation and identification of RRMSCs. **(A)** Isolation of renal resident MSCs (RRMSCs) by fluorescence-activated cell sorting (FACS). **(B)** Oil red O staining and Alizarin Red staining of RRMSCs after the induction of adipogenic and osteogenic differentiation.

Supplementary Table S1 List of primers sequences.

## References

[B1] Basalova N. Sagaradze G. Arbatskiy M. Evtushenko E. Kulebyakin K. Grigorieva O. (2020). Secretome of Mesenchymal Stromal Cells Prevents Myofibroblasts Differentiation by Transferring Fibrosis-Associated microRNAs within Extracellular Vesicles. Cells 9, 1272. 10.3390/cells9051272 PMC7290371 32443855

[B2] Beech R. D. Qu J. Leffert J. J. Lin A. Hong K. A. Hansen J. (2012). Altered Expression of Cytokine Signaling Pathway Genes in Peripheral Blood Cells of Alcohol Dependent Subjects: Preliminary Findings. Alcohol. Clin. Exp. Res. 36, 1487–1496. 10.1111/j.1530-0277.2012.01775.x 22471388 PMC3393821

[B3] Cao H. Chen X. Hou J. Wang C. Xiang Z. Shen Y. (2020). The Shh/Gli Signaling cascade Regulates Myofibroblastic Activation of Lung-Resident Mesenchymal Stem Cells via the Modulation of Wnt10a Expression during Pulmonary Fibrogenesis. Lab. Invest. 100, 363–377. 10.1038/s41374-019-0316-8 31541181

[B4] Devocelle A. Lecru L. François H. Desterke C. Gallerne C. Eid P. (2019). Inhibition of TGF-Β1 Signaling by IL-15: A Novel Role for IL-15 in the Control of Renal Epithelial-Mesenchymal Transition: IL-15 Counteracts TGF-Β1-Induced EMT in Renal Fibrosis. Int. J. Cel Biol. 2019, 1–15. 10.1155/2019/9151394 PMC6642769 31360169

[B5] Estess P. Nandi A. Mohamadzadeh M. Siegelman M. H. (1999). Interleukin 15 Induces Endothelial Hyaluronan Expression *In Vitro* and Promotes Activated T Cell Extravasation through a CD44-dependent Pathway *In Vivo* . J. Exp. Med. 190, 9–20. 10.1084/jem.190.1.9 10429666 PMC2195564

[B6] Fiore P. F. Di Matteo S. Tumino N. Mariotti F. R. Pietra G. Ottonello S. (2020). Interleukin-15 and Cancer: Some Solved and many Unsolved Questions. J. Immunother. Cancer 8, e001428. 10.1136/jitc-2020-001428 33203664 PMC7674108

[B7] Fu C. Lu Y. Williams M. A. Brantly M. L. Ventetuolo C. E. Morel L. M. (2021). Emergency Myelopoiesis Contributes to Immune Cell Exhaustion and Pulmonary Vascular Remodelling. Br. J. Pharmacol. 178, 187–202. 10.1111/bph.14945 31793661 PMC8240454

[B8] Guo S. Smeltz R. B. Nanajian A. Heller R. (2020). IL-15/IL-15Rα Heterodimeric Complex as Cancer Immunotherapy in Murine Breast Cancer Models. Front. Immunol. 11, 614667. 10.3389/fimmu.2020.614667 33628206 PMC7897681

[B9] Höchst B. Mikulec J. Baccega T. Metzger C. Welz M. Peusquens J. (2015). Differential Induction of Ly6G and Ly6C Positive Myeloid Derived Suppressor Cells in Chronic Kidney and Liver Inflammation and Fibrosis. PLoS One 10, e0119662. 10.1371/journal.pone.0119662 25738302 PMC4349817

[B10] Hydes T. Noll A. Salinas‐Riester G. Abuhilal M. Armstrong T. Hamady Z. (2018). IL‐12 and IL‐15 Induce the Expression of CXCR6 and CD49a on Peripheral Natural Killer Cells. Immun. Inflamm. Dis. 6, 34–46. 10.1002/iid3.190 28952190 PMC5818449

[B11] Jiang M. H. Li G. Liu J. Liu L. Wu B. Huang W. (2015). Nestin+ Kidney Resident Mesenchymal Stem Cells for the Treatment of Acute Kidney Ischemia Injury. Biomaterials 50, 56–66. 10.1016/j.biomaterials.2015.01.029 25736496

[B12] Jiao J. Ooka K. Fey H. Fiel M. I. Rahmman A. H. Kojima K. (2016). Interleukin-15 Receptor α on Hepatic Stellate Cells Regulates Hepatic Fibrogenesis in Mice. J. Hepatol. 65, 344–353. 10.1016/j.jhep.2016.04.020 27154062 PMC5048472

[B13] Ke X. Do D. C. Li C. Zhao Y. Kollarik M. Fu Q. (2019). Ras Homolog Family Member A/Rho-associated Protein Kinase 1 Signaling Modulates Lineage Commitment of Mesenchymal Stem Cells in Asthmatic Patients through Lymphoid Enhancer-Binding Factor 1. J. Allergy Clin. Immunol. 143, 1560–1574. e1566. 10.1016/j.jaci.2018.08.023 30194990 PMC6401351

[B14] Kramann R. Schneider R. K. Dirocco D. P. Machado F. Fleig S. Bondzie P. A. (2015). Perivascular Gli1+ Progenitors Are Key Contributors to Injury-Induced Organ Fibrosis. Cell Stem Cell 16, 51–66. 10.1016/j.stem.2014.11.004 25465115 PMC4289444

[B15] Kuppe C. Ibrahim M. M. Kranz J. Zhang X. Ziegler S. Perales-Patón J. (2021). Decoding Myofibroblast Origins in Human Kidney Fibrosis. Nature 589, 281–286. 10.1038/s41586-020-2941-1 33176333 PMC7611626

[B16] Lebleu V. S. Taduri G. O'connell J. Teng Y. Cooke V. G. Woda C. (2013). Origin and Function of Myofibroblasts in Kidney Fibrosis. Nat. Med. 19, 1047–1053. 10.1038/nm.3218 23817022 PMC4067127

[B17] Lebrun A. Lo Re S. Chantry M. Izquierdo Carerra X. Uwambayinema F. Ricci D. (2017). CCR2+monocytic Myeloid-Derived Suppressor Cells (M-MDSCs) Inhibit Collagen Degradation and Promote Lung Fibrosis by Producing Transforming Growth Factor-Β1. J. Pathol. 243, 320–330. 10.1002/path.4956 28799208

[B18] Li J. Tu G. Zhang W. Zhang Y. Zhang X. Qiu Y. (2021). CHBP Induces Stronger Immunosuppressive CD127+ M-MDSC via Erythropoietin Receptor. Cell Death Dis 12, 177. 10.1038/s41419-021-03448-7 33579907 PMC7881243

[B19] Lin S.-J. Chen J.-Y. Kuo M.-L. Hsiao H.-S. Lee P.-T. Huang J.-L. (2016). Effect of Interleukin-15 on CD11b, CD54, and CD62L Expression on Natural Killer Cell and Natural Killer T-like Cells in Systemic Lupus Erythematosus. Mediators Inflamm. 2016, 1–8. 10.1155/2016/9675861 PMC5101392 27847409

[B20] Ma H. Li Y.-n. Song L. Liu R. Li X. Shang Q. (2020). Macrophages Inhibit Adipogenic Differentiation of Adipose Tissue Derived Mesenchymal Stem/stromal Cells by Producing Pro-inflammatory Cytokines. Cell Biosci 10, 88. 10.1186/s13578-020-00450-y 32699606 PMC7372775

[B21] Ma O. K.-F. Chan K. H. (2016). Immunomodulation by Mesenchymal Stem Cells: Interplay between Mesenchymal Stem Cells and Regulatory Lymphocytes. Wjsc 8, 268–278. 10.4252/wjsc.v8.i9.268 27679683 PMC5031888

[B22] Manohar M. Kandikattu H. K. Verma A. K. Mishra A. (2018). IL-15 Regulates Fibrosis and Inflammation in a Mouse Model of Chronic Pancreatitis. Am. J. Physiology-Gastrointestinal Liver Physiol. 315, G954–G965. 10.1152/ajpgi.00139.2018 PMC6336943 30212254

[B23] Ouhtit A. Thouta R. Zayed H. Gaur R. L. Fernando A. Rahman M. (2020). CD44 Mediates Stem Cell Mobilization to Damaged Lung via its Novel Transcriptional Targets, Cortactin and Survivin. Int. J. Med. Sci. 17, 103–111. 10.7150/ijms.33125 31929744 PMC6945551

[B24] Patidar M. Yadav N. Dalai S. K. (2016). Interleukin 15: A Key Cytokine for Immunotherapy. Cytokine Growth Factor. Rev. 31, 49–59. 10.1016/j.cytogfr.2016.06.001 27325459

[B25] Pelekanos R. A. Li J. Gongora M. Chandrakanthan V. Scown J. Suhaimi N. (2012). Comprehensive Transcriptome and Immunophenotype Analysis of Renal and Cardiac MSC-like Populations Supports strong Congruence with Bone Marrow MSC Despite Maintenance of Distinct Identities. Stem Cel Res. 8, 58–73. 10.1016/j.scr.2011.08.003 22099021

[B26] Qi J. Tang X. Li W. Chen W. Yao G. Sun L. (2020). Mesenchymal Stem Cells Inhibited the Differentiation of MDSCs via COX2/PGE2 in Experimental Sialadenitis. Stem Cel Res Ther 11, 325. 10.1186/s13287-020-01837-x PMC7391592 32727564

[B27] Regmi S. Pathak S. Kim J. O. Yong C. S. Jeong J.-H. (2019). Mesenchymal Stem Cell Therapy for the Treatment of Inflammatory Diseases: Challenges, Opportunities, and Future Perspectives. Eur. J. Cel Biol. 98, 151041. 10.1016/j.ejcb.2019.04.002 31023504

[B28] Saldaña L. Bensiamar F. Vallés G. Mancebo F. J. García-Rey E. Vilaboa N. (2019). Immunoregulatory Potential of Mesenchymal Stem Cells Following Activation by Macrophage-Derived Soluble Factors. Stem Cel Res Ther 10, 58. 10.1186/s13287-019-1156-6 PMC6375172 30760316

[B29] Sierra-Parraga J. M. Merino A. Eijken M. Leuvenink H. Ploeg R. Møller B. K. (2020). Reparative Effect of Mesenchymal Stromal Cells on Endothelial Cells after Hypoxic and Inflammatory Injury. Stem Cel Res Ther 11, 352. 10.1186/s13287-020-01869-3 PMC7424997 32787906

[B30] Tang W. Zhang Y. Tang L. Zhang J. Xiong L. Wang B. (2018). Inhibitory Effect of Tranilast on the Myofibroblast Differentiation of Rat Mesenchymal Stem Cells Induced by Transforming Growth Factor-β1 In V-itro. Mol. Med. Rep. 18, 5693–5700. 10.3892/mmr.2018.9588 30365138

[B31] Venkateshaiah S. U. Niranjan R. Manohar M. Verma A. K. Kandikattu H. K. Lasky J. A. (2019). Attenuation of Allergen-, IL-13-, and TGF-α-Induced Lung Fibrosis after the Treatment of rIL-15 in Mice. Am. J. Respir. Cel Mol Biol 61, 97–109. 10.1165/rcmb.2018-0254OC PMC6604217 30702923

[B32] Volarevic V. Gazdic M. Simovic Markovic B. Jovicic N. Djonov V. Arsenijevic N. (2017). Mesenchymal Stem Cell-Derived Factors: Immuno-Modulatory Effects and Therapeutic Potential. Biofactors 43, 633–644. 10.1002/biof.1374 28718997

[B33] Wang Y. Guo X. Jiao G. Luo L. Zhou L. Zhang J. (2019). Splenectomy Promotes Macrophage Polarization in a Mouse Model of Concanavalin A- (ConA-) Induced Liver Fibrosis. Biomed. Res. Int. 2019, 1–12. 10.1155/2019/5756189 PMC6339718 30723740

[B34] Yan J.-J. Ryu J.-H. Piao H. Hwang J. H. Han D. Lee S.-K. (2020). Granulocyte Colony-Stimulating Factor Attenuates Renal Ischemia-Reperfusion Injury by Inducing Myeloid-Derived Suppressor Cells. Jasn 31, 731–746. 10.1681/ASN.2019060601 32132198 PMC7191933

[B35] Yang Y. Lundqvist A. (2020). Immunomodulatory Effects of IL-2 and IL-15; Implications for Cancer Immunotherapy. Cancers 12, 3586. 10.3390/cancers12123586 PMC7761238 33266177

