# Supplementary material for: Monocytic Myeloid-Derived Suppressor Cells Inhibit Myofibroblastic Differentiation in Mesenchymal Stem Cells Through IL-15 Secretion
Source: Front Cell Dev Biol. 2022 Feb 17;10:817402. doi: 10.3389/fcell.2022.817402 (PMC8891503; doi:10.3389/fcell.2022.817402)
Supplement: Supplementary file 1 [file Table1.docx]

**Table S1. List of primers sequences.**

| Target | Primer sequence (5'→3') |
| --- | --- |
| COL1A1 F | GAAACCCGAGGTATGCTTGA |
| COL1A1 R | GGGTCCCTCGACTCCTACAT |
| COL3A1 F | ACCAAAAGGTGATGCTGGAC |
| COL3A1 R | GACCTCGTGCTCCAGTTAGC |
| ACTA2 F | TCTGGACGTACAACTGGTATTG |
| ACTA2 R | GGCAGTAGTCACGAAGGAATAG |
| GAPDH F | AGGTCGGTGTGAACGGATTTG |
| GAPDH R | TGTAGACCATGTAGTTGAGGTCA |
| CD44 F | CACCATTGCCTCAACTGTGC |
| CD44 R | TTGTGGGCTCCTGAGTCTGA |
| ICAM1 F | GTGATGCTCAGGTATCCATCCA |
| ICAM1 R | CACAGTTCTCAAAGCACAGCG |
| VCAM1 F | TTGGGAGCCTCAACGGTACT |
| VCAM1 R | GCAATCGTTTTGTATTCAGGGGA |
