# Supplementary figures and images for: Monocytic Myeloid-Derived Suppressor Cells Inhibit Myofibroblastic Differentiation in Mesenchymal Stem Cells Through IL-15 Secretion
Source: Front Cell Dev Biol. 2022 Feb 17;10:817402. doi: 10.3389/fcell.2022.817402 (PMC8891503; doi:10.3389/fcell.2022.817402)

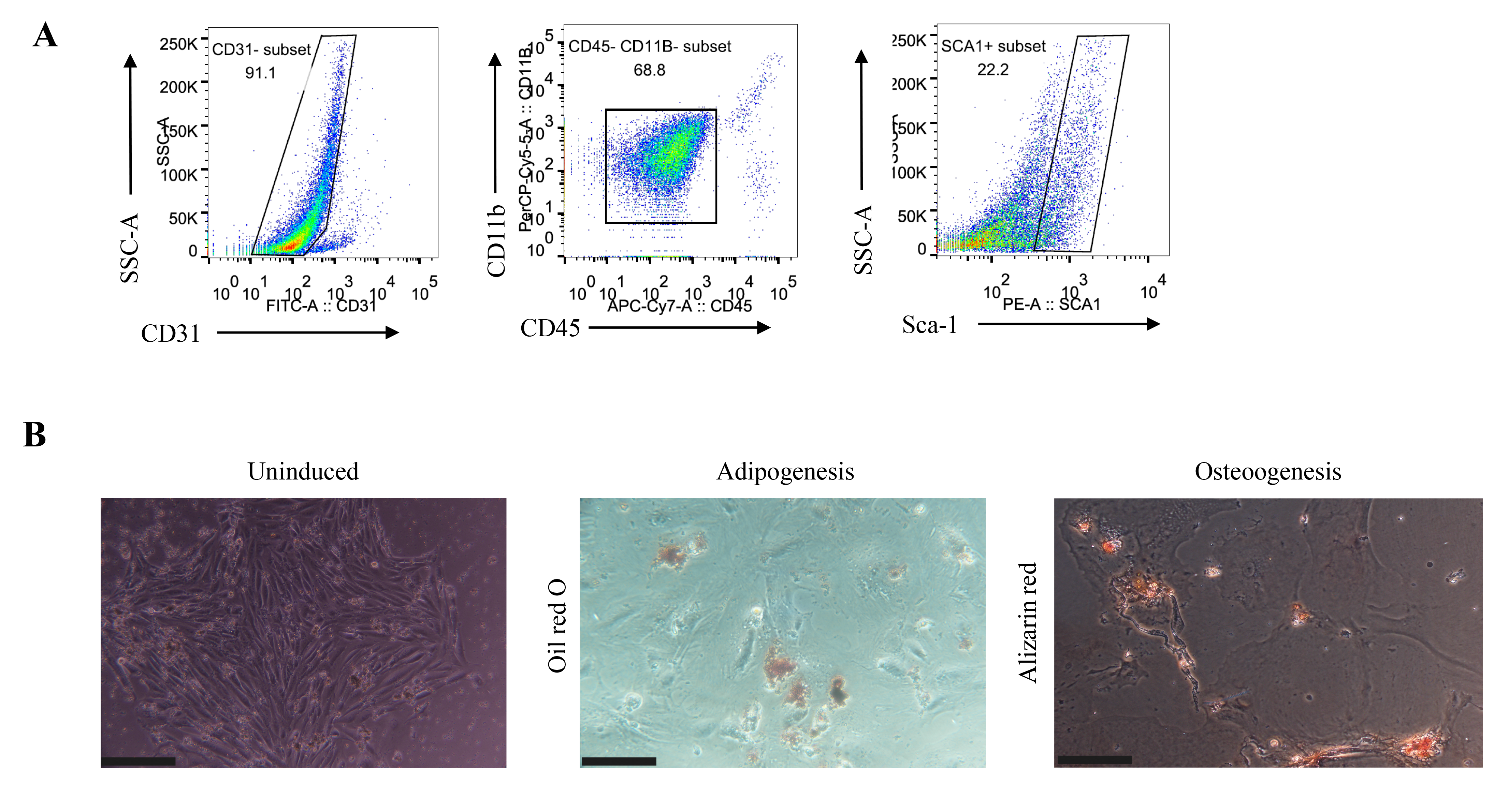

Supplement: Supplementary file 2 [file Image2.tif]

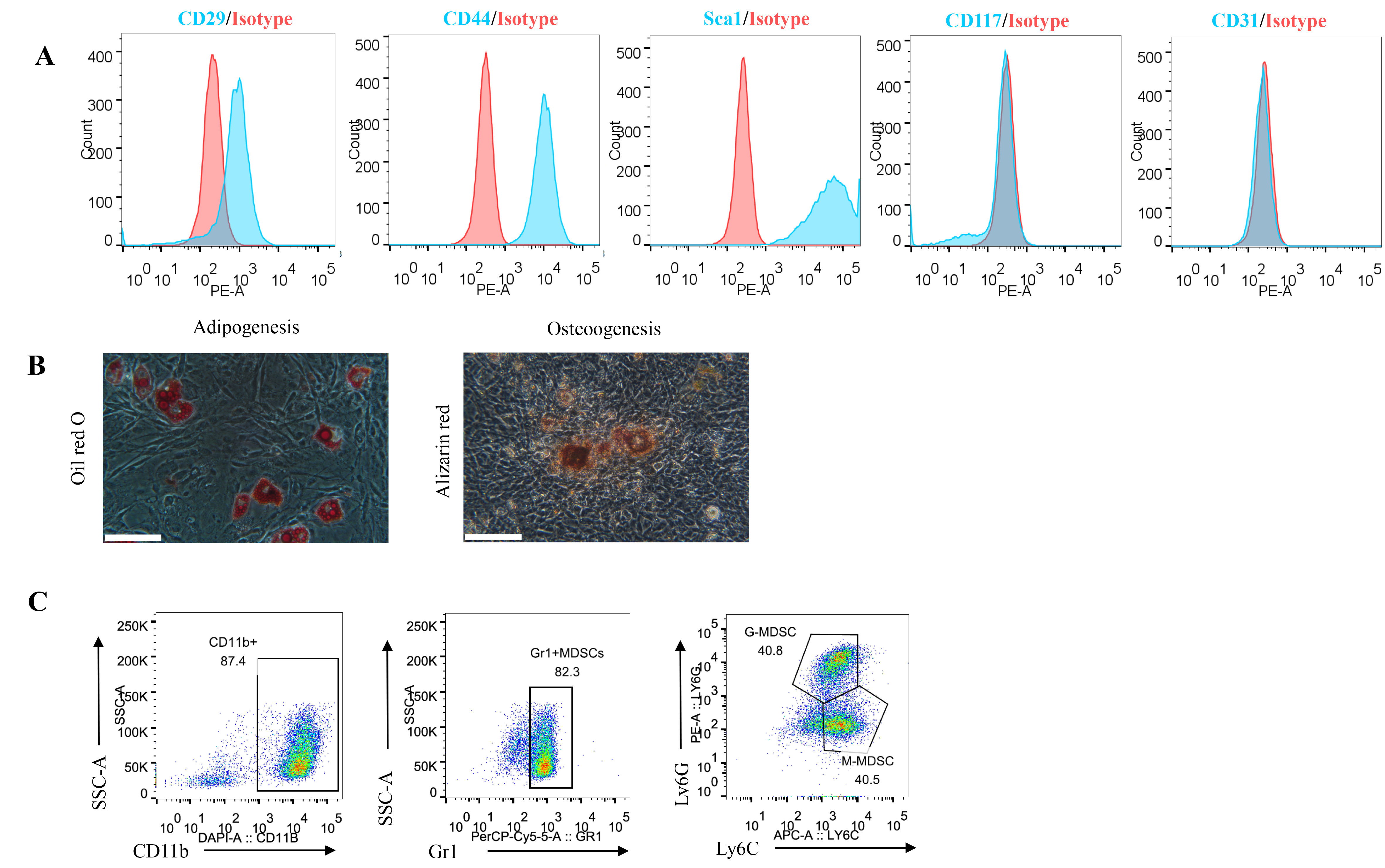

Supplement: Supplementary file 3 [file Image1.tif]
